# Comparison of the Effect of Melatonin Treatment before and after Brain Ischemic Injury in the Inflammatory and Apoptotic Response in Aged Rats

**DOI:** 10.3390/ijms19072097

**Published:** 2018-07-19

**Authors:** Lisa Rancan, Sergio D. Paredes, Cruz García, Pablo González, Cruz Rodríguez-Bobada, Mario Calvo-Soto, Bryan Hyacinthe, Elena Vara, Jesús A. F. Tresguerres

**Affiliations:** 1 Department of Biochemistry and Molecular Biology, School of Medicine, Complutense University of Madrid, 28040 Madrid, Spain; mcruzg@ucm.es (C.G.); mcalvo07@ucm.es (M.C.-S.); bryanhya@ucm.es (B.H.); evaraami@ucm.es (E.V.); 2 Department of Physiology, School of Medicine, Complutense University of Madrid, 28040 Madrid, Spain; spared01@ucm.es (S.D.P.); guerres@ucm.es (J.A.F.T.); 3 Research Unit, Hospital Clínico San Carlos, 28040 Madrid, Spain; pabloantonio.gonzalez@salud.madrid.org; 4 Biomedical Research Foundation, Hospital Clínico San Carlos, 28040 Madrid, Spain; cruzrbg@yahoo.es

**Keywords:** aging, brain, ischemia, melatonin, middle cerebral artery blockade

## Abstract

Aging is associated with an increase in stroke risk. Melatonin, a potent free radical scavenger and broad spectrum antioxidant, has been shown to counteract inflammation and apoptosis in brain injury. However, little is known on the possible protective effects of melatonin in aged individuals affected by brain ischemia. Also, using melatonin before or after an ischemic stroke may result in significantly different molecular outcomes. The objective of the present study was to compare the effects of pre-ischemia vs. post-ischemia melatonin administration in an ischemic lesion in the cortex and hippocampus of senescent Wistar rats. An obstruction of the middle cerebral artery (MCA) to 18-month-old animals was performed. In general, animals treated with melatonin from 24 h prior to surgery until 7 days after the surgical procedure (PrevT) experienced a significant decrease in the levels of tumor necrosis factor-α (TNF-α), interleukin-1β (IL-1β), glial fibrillary acidic protein (GFAP), Bcl-2-associated death promoter (BAD), and Bcl-2-associated X protein (BAX) in both cortex and hippocampus, while hippocampal levels of sirtuin 1 (SIRT1) and B-cell lymphoma 2 (Bcl-2) increased. Treatment of animals with melatonin only after surgery (AT) resulted in similar effects, but to a lesser extent than in the PrevT group. In any case, melatonin acted as a valuable therapeutic agent protecting aged animals from the harmful effects of cerebral infarction.

## 1. Introduction

Stroke is one of the leading causes of death in developed countries [1] causing permanent disability in adults worldwide [2]. The sudden occlusion of a blood vessel by a thrombus or embolism is the most common cause of stroke, resulting in the loss of oxygen and glucose supply to the cerebral tissue. Although stroke is a complex pathological process, increasing evidence shows that ischemic injury coursing with inflammation plays a pivotal role [3]. Cerebral ischemia triggers an ischemic cascade causing irreversible neuronal damage in the ischemic core within minutes of the onset [4]. However, a much larger volume of brain tissue surrounding the ischemic core, known as the penumbra, can be recovered if the blood flow is promptly restored.

Ischemic stroke leads to oxidative stress, microvascular injury, blood-brain barrier dysfunction and post-ischemic inflammation causing ultimately the death of glia, neurons and endothelial cells. The extent of permanent damage depends mainly on the duration and the degree of ischemia and the ability of the brain to repair itself and recover from the insult [4].

Excitotoxicity and oxidative stress activate astrocytes and microglia which react by secreting chemokines, cytokines and matrix metalloproteases (MMP). These molecules lead to upregulation of cell adhesion molecules in endothelial cells, allowing blood-derived inflammatory cells to infiltrate the ischemic area. Neutrophils also secrete cytokines which cause further activation of glial cells. These processes result in neuronal cell death, enhancing brain damage. Increased levels of pro-inflammatory cytokines and decreased anti-inflammatory cytokine production are related to greater damage and poorer clinical outcome. In this regard, it has been observed that interleukin-1β (IL-1β) and tumor necrosis factor-α (TNF-α) appear to intensify cerebral injury [5,6,7], whereas transforming growth factor-β (TGF-β) and IL-10 may be neuroprotective [8,9].

Melatonin (*N*-acetyl-5-methoxytryptamine) is a hormone produced by the pineal gland. It has been shown to have several potential therapeutic benefits, including its potential to counteract age-related alterations [10,11]. Both animal and human studies have shown that its acute toxicity is extremely low. Based on its ability to function as a free radical scavenger, it has been suggested that this indoleamine may act as a neuroprotective agent, protecting the brain from lipid peroxidation and free radical damage induced by reactive oxygen species (ROS) during brain ischemia [12]. This is in agreement with previous results of our group showing that melatonin treatment was able to diminish inflammation and apoptosis after brain stroke [7].

Although significant advance has been made in the understanding of the pathophysiology of cerebral ischemia, therapeutic options for acute ischemic stroke remain very limited [2]. Consequently, successful treatment of acute ischemic stroke remains one of the major challenges in clinical medicine. This is of great significance since the total incidence of stroke is projected to rise substantially over the years as a result of the expanding elderly population. Nonetheless, elderly people have generally been underrepresented in clinical trials, despite age being considered one of the most significant prognostic markers for poor stroke outcome, maybe as a result of the very few experimental studies performed in aged animals, which have created many uncertainties and less optimal medical care for elderly patients. In relation to this, the prescription and use of melatonin as a preventive drug against cerebrovascular accidents in aged individuals is far from being widespread in the clinical context. In addition, using melatonin before or after ischemic stroke may result in significantly different molecular changes, something that needs further clarification. Therefore, the present study aimed to compare the effect of pre-ischemia versus post-ischemia melatonin administration in aged rats subjected to ischemic brain injury.

## 2. Results

In previous studies, we observed that expression of TNF-α, IL-1β, glial fibrillary acidic protein (GFAP), Bcl-2-associated death promoter (BAD) and Bcl-2-associated X protein (BAX) increased significantly after right middle cerebral artery (MCA) occlusion, whereas sirtuin 1 (SIRT1) and B-cell lymphoma 2 (Bcl-2) significantly decreased [7].

**Figure 1:**
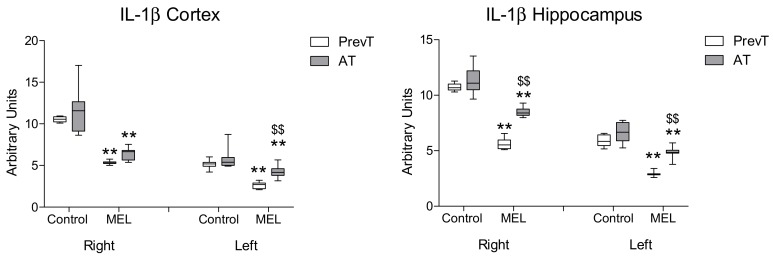
Right and left cortical and hippocampal mRNA expression of IL-1β in 18-month-old rats in control (non-treated) conditions, after treatment with a daily dose of melatonin (10 mg/kg b.w.) from 24 h before until 7 days after surgery (PrevT), and treated only after surgery during 7 days (AT). Each value represents the median, interquartile and full range of five determinations performed in duplicate. ** *p* < 0.01 with respect to the values obtained in the control group; $$ *p* < 0.01 with respect to the values obtained in the PrevT group.

In the present study, both pre- and post-ischemia melatonin administrations were able to decrease IL-1β levels in both hemispheres in a significant manner (*p* < 0.01). In the hippocampus, pre-ischemia melatonin administration showed a significantly higher reduction in IL-1β levels, as compared to the post-ischemia treatment (*p* < 0.01). In cortex, no significant differences were found between the melatonin-treated groups in the ipsilateral (right) hemisphere (Figure 1). However, in the contralateral (left) side, PrevT (pre-ischemia treatment) animals showed significantly (*p* < 0.01) lower levels of this cytokine.

**Figure 2:**
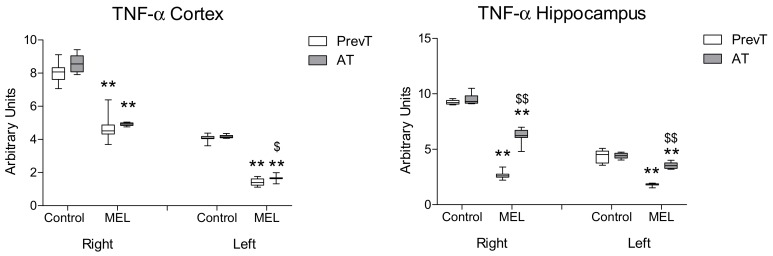
Right and left cortical and hippocampal mRNA expression of TNF-α in 18-month-old rats in control (non-treated) conditions, after treatment with a daily dose of melatonin (10 mg/kg b.w.) from 24 h before until 7 days after surgery (PrevT), and treated only after surgery during 7 days (AT). Each value represents the median, interquartile and full range of five determinations performed in duplicate. ** *p* < 0.01 with respect to the values obtained in the control group; $ *p* < 0.05 with respect to the values obtained in the PrevT group; $$ *p* < 0.01 with respect to the values obtained in the PrevT group.

In the case of hippocampal TNF-α expression, pre-ischemia melatonin administration showed a significantly higher reduction (*p* < 0.01) as compared to the AT group. However, post-ischemia melatonin administration was also able to decrease significantly TNF-α levels in both right and left hippocampus, as compared to their respective controls (Figure 2). A similar effect was observed in the cortex, although here the levels of this pro-inflammatory marker showed a significant reduction (*p* < 0.05) in the left cortex of PrevT animals in comparison with the AT group.

**Figure 3:**
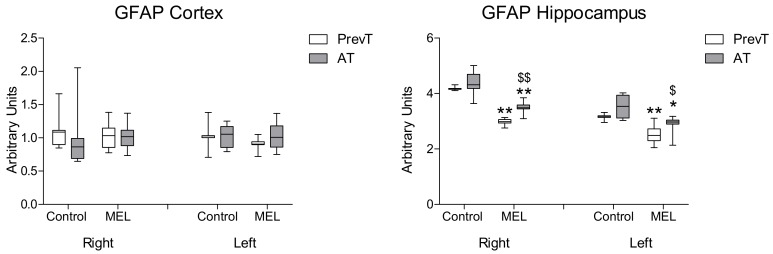
Right and left cortical and hippocampal mRNA expression of GFAP in 18-month-old rats in control (non-treated) conditions, after treatment with a daily dose of melatonin (10 mg/kg b.w.) from 24 h before until 7 days after surgery (PrevT), and treated only after surgery during 7 days (AT). Each value represents the median, interquartile and full range of five determinations performed in duplicate. * *p* < 0.05 with respect to the values obtained in the control group; ** *p* < 0.01 with respect to the values obtained in the control group; $ *p* < 0.05 with respect to the values obtained in the PrevT group; $$ *p* < 0.01 with respect to the values obtained in the PrevT group.

Regarding GFAP (Figure 3), no significant differences were found in cortex with either pre- or post-ischemia treatments. However, post-ischemia melatonin administration was able to decrease significantly GFAP levels in both right (*p* < 0.01) and left hippocampus (*p* < 0.05). The PrevT group experienced a significantly higher reduction in GFAP levels, as compared to the AT treated group. This difference was more significant in the ipsilateral (right) side of the lesion (*p* < 0.01).

**Figure 4:**
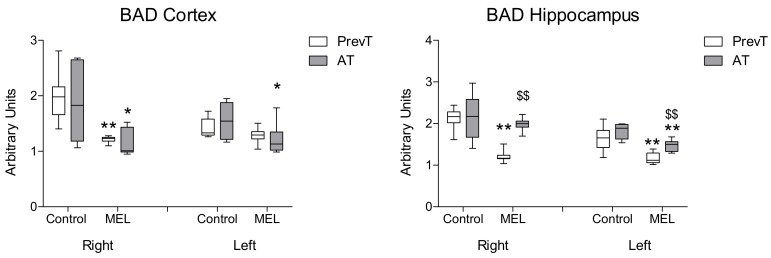
Right and left cortical and hippocampal mRNA expression of BAD in 18-month-old rats in control (non-treated) conditions, after treatment with a daily dose of melatonin (10 mg/kg b.w.) from 24 h before until 7 days after surgery (PrevT), and treated only after surgery during 7 days (AT). Each value represents the median, interquartile and full range of five determinations performed in duplicate. * *p* < 0.05 with respect to the values obtained in the control group; ** *p* < 0.01 with respect to the values obtained in the control group; $$ *p* < 0.01 with respect to the values obtained in the PrevT group.

**Figure 5:**
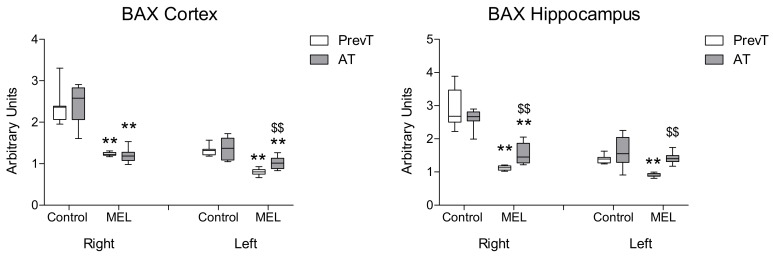
Right and left cortical and hippocampal mRNA expression of BAX in 18-month-old rats in control (non-treated) conditions, after treatment with a daily dose of melatonin (10 mg/kg b.w.) from 24 h before until 7 days after surgery (PrevT), and treated only after surgery during 7 days (AT). Each value represents the median, interquartile and full range of five determinations performed in duplicate. ** *p* < 0.01 with respect to the values obtained in the control group; $$ *p* < 0.01 with respect to the values obtained in the PrevT group.

Melatonin treatment also reduced the expression of pro-apoptotic markers BAD (Figure 4) and BAX (Figure 5). In right and left hippocampus, a significantly higher reduction (*p* < 0.01) in these expressions was found in the PrevT group, as compared to the post-ischemia treatment. Regarding cortex, there were no significant differences between the melatonin-treated groups in BAD cortical levels; in the case of BAX, the pre-ischemia treatment achieved a significantly higher decrease in the contralateral (left) hemisphere (*p* < 0.01).

**Figure 6:**
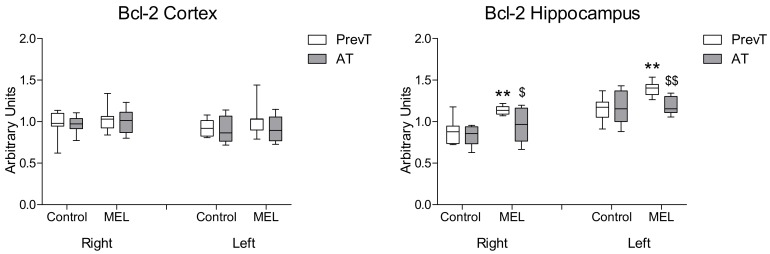
Right and left cortical and hippocampal mRNA expression of Bcl-2 in 18-month-old rats in control (non-treated) conditions, after treatment with a daily dose of melatonin (10 mg/kg b.w.) from 24 h before until 7 days after surgery (PrevT), and treated only after surgery during 7 days (AT). Each value represents the median, interquartile and full range of five determinations performed in duplicate. ** *p* < 0.01 with respect to the values obtained in the control group; $ *p* < 0.05 with respect to the values obtained in the PrevT group; $$ *p* < 0.01 with respect to the values obtained in the PrevT group.

With respect to the anti-apoptotic marker Bcl-2 (Figure 6), no significant differences were found in cortex after melatonin treatment. In hippocampus, the administration of this indoleamine resulted in PrevT values being significantly higher in right (*p* < 0.05) and left (*p* < 0.01) hemispheres than those observed in the AT group.

**Figure 7:**
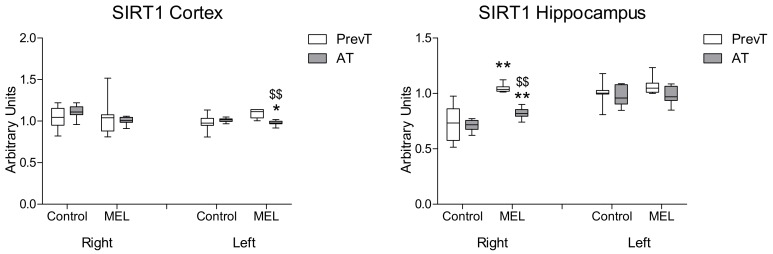
Right and left cortical and hippocampal mRNA expression of SIRT1 in 18-month-old rats in control (non-treated) conditions, after treatment with a daily dose of melatonin (10 mg/kg b.w.) from 24 h before until 7 days after surgery (PrevT), and treated only after surgery during 7 days (AT). Each value represents the median, interquartile and full range of five determinations performed in duplicate. * *p* < 0.05 with respect to the values obtained in the control group; ** *p* < 0.01 with respect to the values obtained in the control group; $$ *p* < 0.01 with respect to the values obtained in the PrevT group.

Regarding SIRT1 expression (Figure 7), both pre- and post-ischemia melatonin treatments were able to increase significantly the levels of SIRT1 (*p* < 0.01) in the right hippocampus, with a greater effect obtained in the PrevT animals (*p* < 0.01). In the left hippocampus and in both right and left cortex, both pre- and post-ischemia melatonin treatments did not increase SIRT1 levels.

## 3. Discussion

Stroke is a cerebrovascular accident that may cause long-term disability and death. Stroke risk factors include aging, diabetes, elevated lipids, smoking and heavy drinking, high blood pressure, coronary artery disease and heart diseases. Ischemic stroke is the most common form, accounting for around 85% of strokes. The effects of ischemic stroke are relatively rapid because the brain does not store glucose and is unable to perform anaerobic metabolism [13]. Within an hour of the ischemic insult, a core of infarction area occurs, surrounded by an oligemic zone called the ischemic penumbra where autoregulation is ineffective. The critical time period during which the brain tissue is at risk is referred to as the “window of opportunity” since, as previously mentioned, the neurological damage caused by ischemia can be partially or fully reversed by reperfusing the ischemic, yet viable, brain tissue [13,14,15,16,17,18].

When ischemia occurs, the mitochondrial membrane potential and the normal proton gradient are disrupted, resulting in excessive ROS generation via electron transfer for ATP synthesis [19]. This process defeats mitochondrial oxidant-scavenging capacity establishing a vicious positive feedback loop in which increased ROS induce more ROS production. ROS also activate inflammatory responses and damage mitochondrial DNA leading to cell swelling and death.

Inflammatory response to brain injury is initiated by the rapid production of several inflammatory mediators. It has been suggested that pro-inflammatory cytokines including TNF-α and IL-1 play a pivotal role in the pathology of ischemia/reperfusion-induced brain injury. Pro-inflammatory cytokines may contribute to brain damage both directly or indirectly [20].

Melatonin is a ubiquitous hormone that is secreted by the pineal gland primarily during darkness. A number of studies suggest that melatonin and its metabolites are highly effective physiological antioxidants and free radical scavengers [21,22]. Many biochemical and histopathological findings have revealed that melatonin exerts neuroprotective effects following experimental and clinical oxidative injury [23,24,25]. Furthermore, lower circulating levels of melatonin exaggerate the oxidative damage to tissues that are subjected to increased oxidative stress [26]. Melatonin exerts anti-inflammatory effects by inhibiting the synthesis of inflammatory cytokines such as IL-1, IL-6 and TNF-α [10,12].

We had previously demonstrated that melatonin, administered in a preventive fashion, was able to protect against brain ischemia/reperfusion-induced injury [7]. Here, this preventive effect was compared to that generated by the post-ischemia administration of melatonin after the induction of a brain ischemic lesion in the cortex and hippocampus of senescent rats. Immediately after surgery, all animals showed limb-use asymmetry and lateralization signs. Although all groups recovered after surgery, we observed that melatonin-treated animals did it faster than non-treated rats. In addition, some of the non-treated animals died in the first 72 h after surgery while animals receiving melatonin survived. However, no significant differences were observed in the mortality rate among groups. It was seen that both types of treatments were able to decrease the inflammatory response, i.e., TNF-α and IL-1β levels, in the ipsilateral and contralateral ischemic areas of both tissues, but more clearly in the PrevT group. Although the effect was more pronounced with that treatment, the findings of the present study also revealed a protective effect of post-ischemia melatonin administration against the aforementioned pro-inflammatory markers, mainly in the penumbra area. These results may be related to melatonin direct ROS-scavenging effects or its ability to regulate lymphocyte migration.

Regarding GFAP, a biomarker synthesized by astrocytes in response to physical or metabolic injuries, including those under pro-inflammatory or oxidative stress conditions, no significant differences were found in the cortex in melatonin-treated rats. Interestingly, in hippocampus a significant decrease was observed in both PrevT and AT groups. This effect was more evident when melatonin administration started 1 day before surgery (PrevT animals). Considering that during ischemia the formation of a colateral circulation that partially irrigates the ischemic lesion occurs, the ability of the penumbral neurons to be reperfused again appears to be evident and melatonin, therefore, may exert its most beneficial effects on them, limiting the extent of the lesion.

Both oxidative stress and inflammatory response can induce cell death. The two processes by which damaged neurons are known to die are necrosis and apoptosis. Apoptotic mechanisms begin within 1 h after ischemic injury, whereas necrosis begins by 6 h after arterial occlusion. This is important because, hypothetically, neuronal death can be prevented by modifying the process responsible for apoptosis. MCA blockade has been shown to cause a significant increase in the expression of pro-apoptotic markers in aged rats [7]. In the present study, this effect was inhibited by the administration of melatonin. Although the effect of pre-ischemia melatonin treatment was generally higher in both hippocampus and cortex, it is interesting to note that similar results were also obtained with post-ischemia treatment. This suggests that melatonin, by reducing the expression of apoptotic markers, could decrease neuronal damage. This would be in agreement with previous studies of other authors describing that the administration of melatonin after head injury prevented both detectable tissue damage and the cognitive deficits that it produced [27,28].

In the case of SIRT1, a protein marker of cell viability, a significant decrease after surgery had been observed in both right and left hippocampus after the MCA occlusion [7]. Like melatonin production, SIRT1 has an association with aging. However, few studies have investigated the relationship between melatonin and SIRT1 with age. Gutierrez-Cuesta et al. investigated the effects of melatonin on pro-survival processes observing that melatonin was able to enhance SIRT1 protein levels, which suggests that melatonin suppressed oxidative stress in SAMP8 mice allowing SIRT1 levels to increase [29]. According to this, in this study we observed that pre-ischemia melatonin administration induced a higher increase in SIRT1 levels, as compared to the post-ischemia treatment in right hippocampus. Nonetheless, and remarkably, in the left hippocampus, no significant differences were found between the pre- and the post-ischemia melatonin treatments. Only a few of the molecular mechanisms involved in sirtuin regulation by melatonin have been addressed in recent studies [30], suggesting that the melatonin membrane receptor pathway may be involved in the upregulation of SIRT1 activity [31]. Moreover, recent studies have shown that SIRT1 activation protects against early brain injury after experimental subarachnoidal hemorrhage in rats [32]. In particular, SIRT1 activation by resveratrol has been reported to reduce brain edema and neuronal apoptosis [33]. Melatonin-induced upregulation of SIRT1 has also been associated with an increase in the anti-apoptotic factor Bcl-2 and a reduction in the pro-apoptotic factor BAX [34], which may be related to the protective effects exerted by melatonin in the animal model used in this study. In fact, numerous findings report a rise in SIRT1 activity in a diversity of cells and animal models after melatonin treatment, with the exception of some tumor cells, where the effect is inhibitory [30].

The clinical outcomes resulting from cerebral ischemia are wide-ranging and include from short-term effects such as death, hypoxic-ischemic encephalopathy and seizures, to long-term ones including cerebral palsy and sensory, cognitive, developmental, neuroendocrine and behavioral deficits which are permanent and currently incurable. Little is known, however, on the possible protective effects of melatonin in elderly individuals affected by cerebral ischemia. For this reason, a possible strength of this study appears to be the use of aged animals in the ischemic brain injury performed experimentally after MCA occlusion. Nonetheless, a limitation is that neurological or behavioral deficits were not evaluated. However, a number of studies have reported that melatonin treatment shows a suitable profile to prevent and/or ameliorate these deficits, including spatial learning and memory performance, severe loss of pyramidal neurons, preservation of cytoarchitectural characteristics in prefrontal cortex and hippocampal CA1 pyramidal neurons, and a better display of place learning and working memory [35]. Further investigation including the use of immunofluorescence, immunohistochemical, and Western-blot techniques, together with the evaluation of neuronal post-treatment cell death/survival, neuroinflammatory astrocyte and microglial changes, as well as performing neurological and behavioral tests, are needed to elucidate the exact role of melatonin in the aging animal model used in the present study.

## 4. Materials and Methods

### 4.1. Animals

Eighteen-month-old male Wistar rats obtained from Harlan Ibérica (Barcelona, Spain) were housed under standard conditions with a 12/12 h light/dark photoperiod. Access to standard rodent chow (A04; Panlab, Barcelona, Spain) and water was permitted ad libitum. Animals were subjected to a blockade of the right MCA as a model of ischemic brain injury and divided into 3 groups: Control (non-treated), treated with a daily dose of melatonin (10 mg/kg b.w.) from 24 h before until 7 days after surgery (pre-ischemia treatment, PrevT), and treated only after surgery during 7 days (post-ischemia treatment, AT). The study was approved by the Ethical Committee of the Complutense University of Madrid (27 January 2011, Madrid, Spain) in accordance with the European directive on the protection of animals used for scientific purposes (2010/63/EU).

### 4.2. Surgical Procedure

The surgery was carried out following a protocol previously described [36]. Briefly, after premedication (fentanyl and medetomidine, 0.3/0.3 mg/kg b.w.), animals were intubated and maintained under isoflurane anesthesia. Cervical ventral area was shaved and animals were placed in supine position with the forelimbs open. The surgical field was prepared and sterilized. A midline incision from the laryngeal area up to 2 mm cranially to the xiphoid process was made and lengthened 1.5 cm to the right. The right external carotid artery was exposed, carefully separated from the soft tissues of the area, and ligated with a silk 3/0 suture placed caudally to the dissected portion. A microvascular clip was then placed cranially to the ligature. The carotid was opened in the middle point between the ligature and the microvascular clip with a 30 g needle. A 5/0 nylon filament was inserted up to the microvascular clip, which was removed to allow filament insertion into its final position. After assessing that the portion of filament introduced into the carotid artery was correctly positioned, a ligature was placed cranially to the site where the filament had been inserted. Absence of vascular bleeding was then checked and suture in layers (continuous subcutaneous–vicryl 3/0– and skin with interrupted stitches–vicryl or silk 3/0 3/0–) was performed. Isoflurane supply was interrupted, anesthesia was reversed with atipamezole (0.3 mg/kg b.w.) and a broad-spectrum antibiotic (enrofloxacin injectable solution) and an opioid analgesic (buprenorphine) were supplied. Analgesia was repeated at 12 and 24 h after surgery. Throughout the surgical procedure and in the post-surgical recovery, the animals were placed on a heating blanket.

### 4.3. Administration of Melatonin

Melatonin (Actafarma, Madrid, Spain) was dissolved in absolute ethanol, diluted with water to a final concentration of 0.1%, and added to the drinking water at a dose allowing the supply of 10 mg/kg b.w./day in 25–30 mL. The dose of 10 mg/kg b.w. melatonin has been extensively used in scientific literature and it corresponds approximately to a dose of 1.62 mg/kg b.w. in humans, i.e., around 100 mg/day for a 60 kg person [37]. Previous studies observed that the daily administration to adults of doses up to 300 mg of melatonin produced no significant adverse reactions [38]. Moreover, follow-up studies of more than 10 years have reported no side effects [39]. Eighteen-month-old animals (treated groups) started the supply of this melatonin solution 24 h before (PrevT) or immediately after (AT) surgical procedure and during 7 days of post-treatment period until sacrifice. Drinking water with 0.1% ethanol only was available for the non-treated group. At the end of the treatment period, animals were sacrificed by decapitation and cortex and hippocampus of both brain hemispheres were carefully dissected. Tissues were immediately frozen in liquid nitrogen and kept at −80 °C until analyzed.

### 4.4. RNA Isolation and RT-PCR

mRNA expression of IL-1β, TNF-α, BAD, BAX, GFAP, Bcl-2 and SIRT1 was measured by means of reverse transcription-polymerase chain reaction (RT-PCR). RNA was isolated from cortex and hippocampus samples using the TRI Reagent Kit (Molecular Research Center, Inc., Cincinnati, OH, USA), following the manufacturer’s protocol. The purity of the RNA was estimated with 1% agarose gel electrophoresis, and the RNA concentration and purity were determined with spectrophotometry (260/280 nm). Reverse transcription of 2 µg of RNA for cDNA synthesis was performed using the Reverse Transcription System (Promega, Madison, WI, USA), and a pd(N) 6 random hexamer. RT-PCR was performed using an Applied Biosystems 7500 Fast apparatus with the SYBR Green PCR Master Mix (Applied Biosystems, Warrington, UK) and 300 nM concentrations of specific primers (Table 1). RT-PCR amplifications were performed as follows: 50 °C for 2 min, 95 °C for 10 min, followed by 40 cycles of 95 °C for 15 s, 60 °C for 1 min, 95 °C for 15 s, 60 °C for 30 s and 95 °C for 15 s. Normalization of cDNA loading in the PCR was performed using the amplification of 18S ribosomal RNA. Relative changes in gene expression were calculated using the 2^−ΔΔ*C*t^ method.

### 4.5. Statistical Analysis

Data are expressed as median, interquartile and full range of the number of determinations carried out in duplicate. The results were analyzed by ANOVA followed by Fisher’s test. Statistical analyses were carried out using the SPSS 24.0 computer program (SPSS, Chicago, IL, USA). The level of statistical significance was established at *p* < 0.05.

## 5. Conclusions

Melatonin treatment may mediate neuroprotection against ischemic brain injury by limiting the consequential inflammatory and apoptotic responses in both brain hemispheres. Although the effects of melatonin were found to be more evident when treating animals in a preventive way, post-ischemia melatonin administration also showed a significant reduction in inflammation and apoptosis, as compared to the non-treated groups. Thus, melatonin appears as a valuable therapeutic agent that may protect the elderly from the damaging effects of stroke, either before or after injury occurrence, with potential actions in preventing the extent of the lesion.

## Figures and Tables

**Table 1:** Primers used in RT-PCR experiments. 18S was used as a housekeeping gene to compare the samples.

Marker	Primers	Sequence (5′–3′)
18S	Forward	GGTGCATGGCCGTTCTTA
	Reverse	TCGTTCGTTATCGGAATTAACC
IL-1β	Forward	TGTGATGAAAGACGGCACAC
	Reverse	CTTCTTCTTTGGGTATTGTTTGG
TNF-α	Forward	ATGAGAAGTTCCCAAATGGC
	Reverse	CTCCACTTGGTGGTTTGCTA
BAD	Forward	GCCCTAGGCTTGAGGAAGTC
	Reverse	CAAACTCTGGGATCTGGAACA
BAX	Forward	GTGAGCGGCTGCTTGTCT
	Reverse	GGTCCCGAAGTAGGAGAGGA
GFAP	Forward	ACAGACTTTCTCCAACCTCCAG
	Reverse	CCTTCTGACACGGATTTGGT
Bcl-2	Forward	CAGGTATGCACCCAGAGTGA
	Reverse	GTCTCTGAAGACGCTGCTCA
SIRT1	Forward	TCGTGGAGACATTTTTAATCAGG
	Reverse	GCTTCATGATGGCAAGTGG
